# Adsorption of Fibronectin Fragment on Surfaces Using Fully Atomistic Molecular Dynamics Simulations

**DOI:** 10.3390/ijms19113321

**Published:** 2018-10-25

**Authors:** Evangelos Liamas, Karina Kubiak-Ossowska, Richard A. Black, Owen R.T. Thomas, Zhenyu J. Zhang, Paul A. Mulheran

**Affiliations:** 1 School Chemical Engineering, University of Birmingham, Edgbaston, Birmingham B15 2TT, UK; e.liamas@leeds.ac.uk (E.L.); o.r.t.thomas@bham.ac.uk (O.R.T.T.); 2 Department of Chemical and Process Engineering, University of Strathclyde, James Weir Building, 75 Montrose Street, Glasgow G1 1XJ, UK; karina.kubiak@strath.ac.uk; 3 Department of Biomedical Engineering, University of Strathclyde, 106 Rottenrow, Glasgow G4 0NW, UK; richard.black@strath.ac.uk

**Keywords:** NAMD, self-assembled monolayers, SAMs, protein adsorption, explicit solvent

## Abstract

The effect of surface chemistry on the adsorption characteristics of a fibronectin fragment (FNIII^8–10^) was investigated using fully atomistic molecular dynamics simulations. Model surfaces were constructed to replicate self-assembled monolayers terminated with methyl, hydroxyl, amine, and carboxyl moieties. It was found that adsorption of FNIII^8–10^ on charged surfaces is rapid, specific, and driven by electrostatic interactions, and that the anchoring residues are either polar uncharged or of opposing charge to that of the targeted surfaces. On charged surfaces the presence of a strongly bound layer of water molecules and ions hinders FNIII^8–10^ adsorption. In contrast, adsorption kinetics on uncharged surfaces are slow and non-specific, as they are driven by van der Waals interactions, and the anchoring residues are polar uncharged. Due to existence of a positively charged area around its cell-binding region, FNIII^8–10^ is available for subsequent cell binding when adsorbed on a positively charged surface, but not when adsorbed on a negatively charged surface. On uncharged surfaces, the availability of the fibronectin fragment’s cell-binding region is not clearly distinguished because adsorption is much less specific.

## 1. Introduction

Biomaterials are generally defined as materials that interact with living matter and can be used to construct tissue and organs in order to replace or augment the function of their predecessors [1]. Upon exposure to biological fluids the surface of a biomaterial is covered with a dynamic layer of host proteins that mediates the cell–biomaterial interactions through receptors present on the cell membrane [2]. Consequently, the protein–surface interaction plays a significant role in determining whether the biomaterial will be accepted or cause an inflammatory reaction that could lead to rejection of the implant [3,4]. Therefore, a molecular-scale insight on the protein adsorption processes on a range of surfaces with different characteristics underpins the design of biomaterials with enhanced biocompatibility. 

Fibronectin, a large glycoprotein that is found predominantly in the extracellular matrix and the plasma [5], is one of the most important proteins that mediate the biomaterial–cell interaction [6,7]. It is a main cell receptor that influences processes such as cell adhesion and differentiation, while it is also involved in applications such as inflammation and wound repair [7]. It has a molecular weight of approximately 440 kDa and consists of two similar chains held together by a couple of disulphide bonds at the C-terminus. Fibronectin contains a cell-recognition sequence that allows it to bind specifically with integrins on the surface of cells. It is composed of the cell binding and the synergy domains which respectively contain the amino acid sequences Arg–Gly–Asp (RGD) and Pro–His–Ser–Arg–Asn (PHSRN). The domains are situated in the FNIII^10^ and FNIII^9^ modules that can undergo reversible unfolding as a mechanism of elasticity to promote cell-binding [8]. In order for the cell to bind fibronectin, it is crucial that the cell-binding domain remains exposed to solvent after adsorption, because mismatched conformation or denaturation of the protein will inhibit binding to cells [9].

One of the most important factors that affects protein–surface interactions is the surface chemistry of a biomaterial, which can be hydrophobic or hydrophilic, positively or negatively charged. As fibronectin is a polyampholyte (contains both cationic and anionic groups), each of the aforementioned surfaces would have a different level of impact on its conformation upon adsorption. A very convenient method to engineer surfaces with the desired chemical properties involves the use of self-assembled monolayers (SAMs), which are thin films of ordered molecular assemblies formed by spontaneous adsorption of organic molecules on surfaces [10,11,12]. SAMs have been widely used in the past as effective model surfaces for studying protein adsorption [13,14,15,16,17,18]. It has been found that while proteins adsorb strongly on hydrophobic surfaces, this causes structural deformation of the protein which can affect its ability to bind cells [14,15,16]. In contrast, adsorption on hydrophilic surfaces is weaker, but the adsorbed proteins maintain their structural integrity and cell binding ability.

Computational modelling is playing a major role in advancing our understanding of protein adsorption because it provides an insight into the adsorption pathways and the individual interactions between proteins and surfaces. Several computational studies on the effect of surface charge, hydrophobicity, and ions on protein adsorption have been reported [19,20,21,22,23,24,25,26,27,28]. Electrostatic interactions play a key function in driving the proteins onto charged surfaces [19,22,23,28]; solvent ions are important here, because they screen the electric field, thereby promoting or inhibiting protein adsorption [21,23,26]. For adsorption on hydrophobic surfaces, the interplay between the hydrophobic surface and hydrophobic patches on the protein determines the affinity of the protein towards the surface [19,25]. 

Fibronectin adsorption has been mostly studied using Monte Carlo or implicit solvent molecular dynamics (MD) simulations [29,30,31,32]. Adsorption on hydroxyapatite surfaces is driven by electrostatic interactions, while FNIII^10^ undergoes two stages of adsorption—pre-adsorption driven by Coulombic interactions, followed by post-adsorption when the driving force shifts from Coulombic to van der Waals interactions, while the binding site remains exposed [29,32]. Other studies report greater accessibility of FNIII^7–10^ cell-binding domains after adsorption on positively cf. negatively charged surfaces [30,31], and that adsorption on hydrophobic surfaces denatures the protein, leading to loss of the protein’s ability to bind cells [30,31]. 

Continuing developments in computing processing power allow ever-more detailed simulations of larger systems over longer periods. While fully atomistic MD simulations of full-length large proteins, such as fibronectin, remains a challenging task due to the lack of a fully resolved protein structure and the required processing power, investigation of individual domains will shed light on their particular functions and roles in various processes, including binding to cells. In the present work, fully atomistic MD simulations were employed to systematically investigate the adsorption pathways of FNIII^8–10^ during the initial stages of anchoring, in order to identify the properties of anchoring residues on each surface, and study the exposure of the cell-binding domain upon adsorption. Protein trajectories were analysed to identify the driving forces during adsorption; the impact of the adsorption on the structural integrity of the protein; and availability of the cell-binding domain after adsorption. Although the study depicts the initial stages of adsorption, it is widely accepted that the change in protein orientation at a surface is significantly faster than the conformational change [29,33,34]. Therefore, it is expected that the subsequent conformation changes will not change the orientation of the protein fragment on the surface significantly, which has been confirmed in the past during the adsorption of FNIII^7–10^ [31,35,36].

## 2. Results

### 2.1. Protein Structural Features

**Figure 1:**
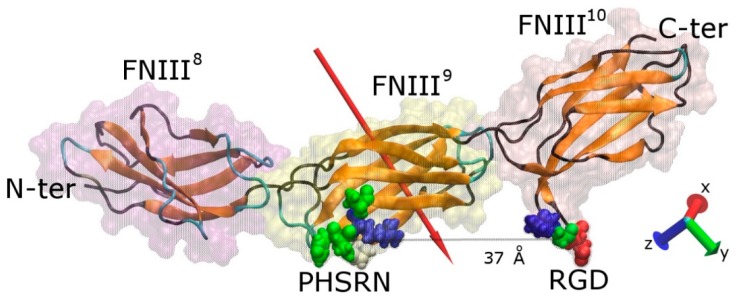
Graphical representation of a FNIII^8–10^ fragment of fibronectin. The secondary structure (colour-coded; orange, teal, and black represent β-sheets, loops, and coil, respectively) along with the protein “ghost” surface of the modules that comprise the fragment are visible. The cell binding (RGD) and synergy (PHSRN) sites, as well as the distance between them, are shown, and the red arrow indicates the dipole moment of the fragment. The amino acids shown are colour-coded with blue, red, white, and green representing positively charged, negatively charged, hydrophobic, and polar uncharged, respectively. For sake of clarity, the water molecules are not shown.

The protein fragment FNIII^8–10^ consists of 274 amino acids (4184 atoms) and has overall dimensions of ~115 × 23 × 23 Å. Modules FNIII^10^ and FNIII^9^ contain the cell-binding and the synergy regions, respectively, which are on the same side of the protein fragment (Figure 1). The RGD motif is recognised by integrins on the surface of cells, whereas the synergy site (PHSRN) determines the specificity and the affinity of integrin binding [7]. The precise conformation of fibronectin will determine which integrin (α_5_β_1_ or α_ν_β_3_) will bind with it [37]. The distance between the two sites is approximately 37 Å when the fragment is not deformed, while the RGD site protrudes approximately 10 Å from fibronectin’s surface and promotes the interaction with integrins. The RGD motif is the most extensively studied cell-binding sequence, being present in matrix molecules such as vitronectin, fibronectin, laminin, and collagen. As such, it has been used as a strategy to promote cell binding to surfaces on which peptides containing this sequence have been adsorbed or chemically immobilised [38]. The RGD motif contains an Arg residue (positively charged) and an Asp residue (negatively charged) lending an overall charge of zero e, whereas the PHSRN site contains a single Arg residue conveying an overall positive charge of +1 e. Both sites are hydrophilic, with hydropathy indices of −8.4 and −13.6 for RGD and PHSRN respectively [39]. The overall charge of the fragment is −5 e distributed unequally among the three modules—module FNIII^8^ has an overall charge of −4 e, FNIII^9^ of −1 e, while FNIII^10^ is neutral (Table S1). The uneven distribution of charged residues results in an overall dipole moment of approximately 750 D, which is oriented almost perpendicular to the long axis of the protein, pointing towards the side that contains the cell-binding and synergy regions, consistent with previous studies [30].

### 2.2. Simulation in Water 

In order to bind successfully with the cell receptors, the cell-binding region must be exposed and the distance between RGD and PHSRN must remain unaffected. To establish whether the FNIII^8–10^ fragment maintains its integrity during the simulations, it was placed in a simulation box filled with water molecules, Na^+^ and Cl^−^ ions, and a trajectory simulated for 100 ns. The root-mean-square deviation (RMSD), calculated in reference to protein structure at *t* = 0 ns, for each individual module remain constant throughout the simulation, confirming that the fibronectin modules maintain their structural integrity (Figure S1). 

**Figure 2:**
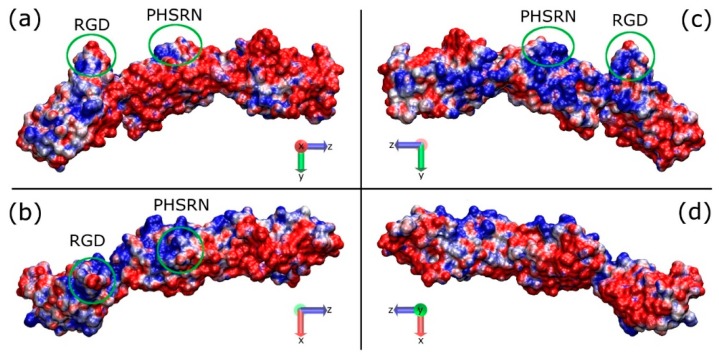
Graphical representations of the FNIII^8–10^ domain showing the electrostatic properties (APBS) along the long axis of the protein; (**a**,**c**) the two sides of the protein on the y-z plane, (**b**,**d**) the two sides of the protein on the x-z plane. The sides from a to d are referred to as FNa, FNb, FNc, and FNd, respectively. The areas in the green circles indicate the RGD and PHSRN sites, which are facing up in (**a**) and (**c**), pointing out from the page in (**b**), and into the page in (**d**). Red and blue indicate the negatively and positively charged domains, respectively.

There is some notable fluctuation with the RMSD of the overall fragment, which could be attributed to the bends between adjacent modules. The measured distance between the RGD and PHSRN sites slowly decreases from 37 Å to a final value between 30 and 35 Å (Figure S1). Due to a heavy bend between the ninth and tenth modules of the fragment structure at around 70 ns into the simulation, the structure of the fragment at *t* = 60 ns was chosen as the initial structure for all the adsorption simulations. It is also found that the core structures of the modules maintain their integrity, whilst the loops between subsequent β-strands showed twists and bends. The distribution of charged amino acids is not regular, with the hydrophobic residues gathered around the core of the protein and the hydrophilic residues exposed to the solvent. A positively charged patch presents at the side of the RGD and PHSRN sites (Figure 2). 

### 2.3. Adsorption on Positively Charged Surface

**Figure 3:**
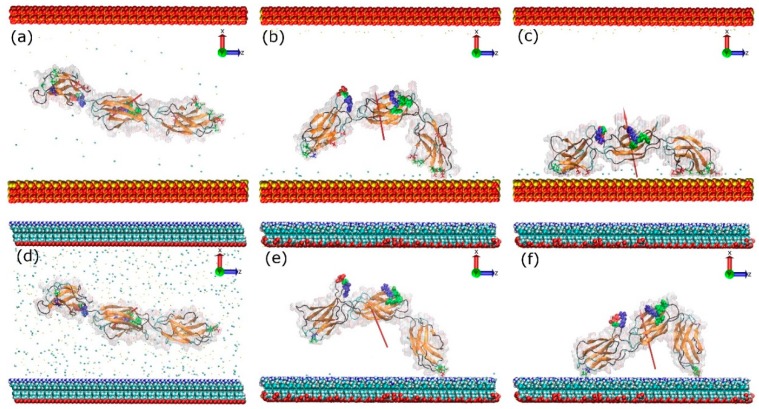
Adsorption process of FNIII^8–10^ on the model silica (top) and amine surface (bottom): *t* = 0 ns (**a**,**d**), anchoring event (**b**,**e**), end of simulation (**c**,**f**). The red arrows indicate the dipole moment of the protein, while the colour-coding is the same as that used in Figure 1. The water molecules are not shown.

The key events of the FNIII^8–10^ fragment adsorption process onto positively charged surfaces, which include a model silica surface (with exposed silicon ions that renders the surface positively charged) and an amine (–NH_3_^+^) surface, are illustrated in Figure 3. Due to an increased number of charged species, the surface charge density of the amine surface is much greater than that of silica, which results in a greater electric field. To obtain similar electric field in both systems additional Na^+^ and Cl^−^ ions were added to the amine surface condition. Due to the polarising effect created by the charged surfaces in both systems, the Na^+^ and Cl^−^ ions are driven to the oppositely charged surfaces and partially screen the force field beyond the Debye length. Measurements of the dipole moment of the protein showed that to match the conditions in the silica system plus 0.05 M NaCl required the use of 0.80 M of NaCl in the amine system. In both systems, the FNc (Figure 2) side of the protein was chosen to face the positively charged surface as the initial configuration, where the cell binding site is on the side of the protein and not directly facing the surface. During the first few nanoseconds, the FNIII^8–10^ fragment quickly rotates along its long axis and aligns its dipole moment with the electric field imposed by the charged surfaces. Subsequently, the protein is attracted towards the positively charged surfaces, starting with the FNIII^8^ module and followed by the FNIII^10^.

Due to the design of the silica surface, the overall charge of FNIII^8–10^, and the 3D periodicity of the simulation cell, the silica system was used to study adsorption on a theoretical positively charged surface. On this system, the initial anchoring occurs after just 3.1 ns with Ser1261 (polar, neutral, and slightly hydrophilic). Following the anchoring event, more residues in the same module adsorbed onto the surface, namely Asp1263 and Glu1312 (both negatively charged). The other end of the fragment approached and anchored on the surface with residue Thr1509 at 23.3 ns. Normally, threonine (Thr) is polar, neutral, and slightly hydrophilic. However, Thr1509 is a C-terminal residue and, therefore, it has a negative charge. At the end of the simulation, nineteen residues were found to adsorb on the silica surface (Table 1). Of these adsorbed residues, six are polar neutral, four are hydrophobic, eight are negatively charged, and one is positively charged. 

**Figure 4:**
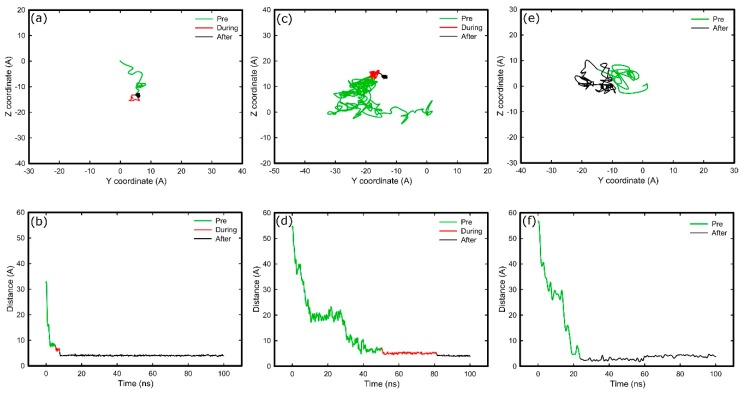
Diffusion of residues over the surface (top), and distance of residues perpendicular to the surface over time (bottom) for; anchoring residue of FNIII^8^ in amine system (Glu1312) (**a**,**b**), anchoring residue of FNIII^10^ in amine system (Thr1509) (**c**,**d**); and anchoring residue of FNIII^10^ in silica system (Thr1509) (**e**,**f**). Green, red, and black respectively illustrate the trajectories before, during, and after anchoring.

A similar adsorption process was observed with the amine surface. After a quick rotation and alignment of the dipole moment, module FNIII^8^ of the fragment was anchored onto the amine surface with Glu1312 (negatively charged) at 5.3 ns. Subsequently, the other end of the protein was anchored at 50.5 ns with Thr1509 due to the electrostatic attraction. In contrast to the silica surface, only the anchoring residues adsorbed onto the amine surface, which is probably caused by a strongly bound layer of water and ions on this surface. The initial rotation resulted in the cell-binding region to remain exposed to the solution in both systems (Figure 3c and Figure 4f). As expected, the electrostatic maps (Figure S2) reveal that the side of the protein contacting the positively charged surfaces is negatively charged. Moreover, a greater fraction of residues belongs to FNIII^8^ than the FNIII^10^ module. Because significantly more residues were found to adsorb on the silica cf. amine-terminated surface, the contact area between protein and silica surface increased accordingly.

Figure 4 shows the trace of residues responsible for anchoring, and their distance to the surface, in both simulation systems. For the amine system, the mobility of the anchoring residue decreases when it reaches the surface (red trace of Figure 4 plots). Subsequently, the residue overcomes a barrier and strongly interacts with the surface, as is reflected by the reduced mobility (black trace of Figure 4 plots). This barrier is probably linked to strongly bound layer of water and ions on the amine surface. Such a barrier was not observed in the silica system (Figure 4e,f), from which we deduce it is either absence or that the anchoring residue was not able to penetrate it. It is worth emphasising that after anchoring on silica, the protein remained mobile along the surface (black trace of Figure 4e), which suggests weaker binding cf. that with the amine surface.

Right after the initial anchoring, there are approximately five hydrogen bonds developed between the amine surface and FNIII^8^ module (Figure S3). Around 50 ns, when module FNIII^10^ anchored the surface, the number of bonds increased to around seven. Eventually, by the end of the simulation there are approximately twelve hydrogen bonds between protein and surface.

### 2.4. Adsorption on Negatively Charged Surface

**Figure 5:**
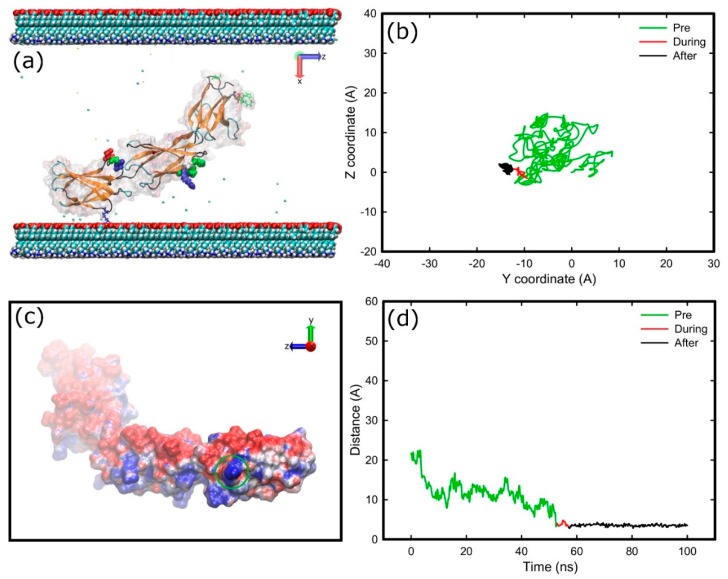
Adsorption of FNIII^8–10^ onto carboxyl surface; (**a**) snapshot at the end of simulation, (**b**) diffusion of the anchoring residue over the surface, (**c**) electrostatic representations (APBS) of the FNIII^8–10^ fragment as viewed from the carboxyl surface on the final stage of adsorption, and (**d**) distance of the anchoring residue perpendicular to the surface over time. The areas inside the green circles indicate the areas that are in contact with the surface. The deep cueing option of VMD is being used to indicate the distance of each atom from the surface. The colour-coding is the same as previously, while surface atoms and water molecules are not shown for sake of clarity.

Because the protein fragment has a negative overall charge, the electric field implemented in the system would force it towards the positively charged surface. In order to promote the adsorption onto the negatively charged surface, the electric field was further screened with the addition of 1 M NaCl (*cf.* 0.8 M for adsorption onto the positively charged surface). The FNa side of the protein was used to face the carboxyl surface as the initial configuration (Figure 2). In this configuration the cell binding site is on the side of the protein, and not directly facing the surface. In contrast to adsorption onto the positively charged surfaces, where the electric field forces the fragment to rotate and adsorb onto the amine-terminated surface, now FNIII^8–10^ undergoes more Brownian-like motion due to more effective screening of the electric field above the surface. The fragment approaches the surface several times without anchoring, because there is no specific preferred orientation, until it anchors with Lys1469 (positively charged) after 53 ns (Figure 5a). It appears that Lys1469, after a weak initial binding, penetrates a barrier (Figure 5d) and binds firmly onto the surface. After anchoring, Lys1469 was immobilised on the surface (Figure 5b). Residue Lys1469 was the only residue that was adsorbed onto the carboxyl surface, which could be attributed to the absence of positively charged residues from the contact area that would be able to penetrate the barrier (Figure 5c).

### 2.5. Adsorption on Hydrophobic Surface

Following adsorption on charged surfaces, we studied the adsorption of the fragment onto uncharged surfaces. Initially, FNIII^8–10^ was inserted between a methyl and a hydroxyl surface (methyl-hydroxyl system) and was let to run its trajectory (Figure S4). The initial orientation of the protein was with side FNa (Figure 2) facing the methyl surface. In the absence of an electric field, the protein freely diffused until 35 ns when Thr1431 (polar, uncharged) from FNIII^10^ module anchored to the methyl surface. Of the total seven residues (Table 1) in contact with the methyl surface, three are hydrophobic, three are polar uncharged, and one is positively charged. At the final stage of adsorption, the fragment had a “head-on” conformation, while the cell-binding region remained exposed to the solution; mobility was little affected by anchoring (Figure S4). 

**Figure 6:**
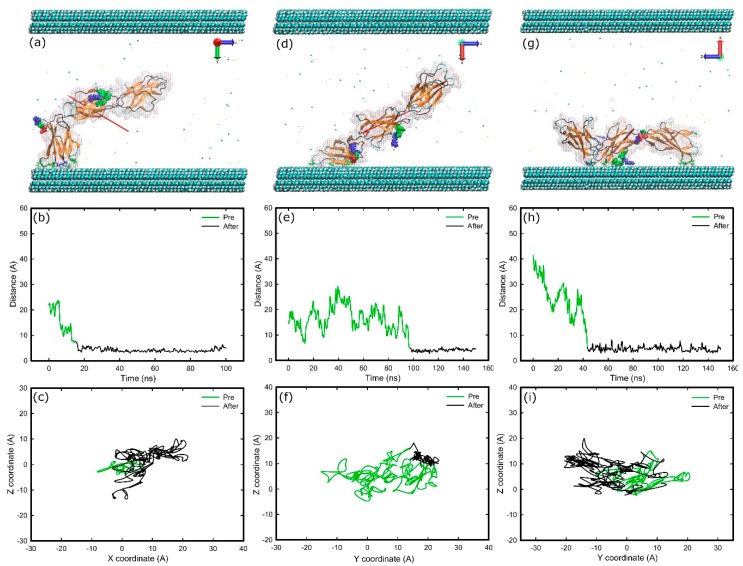
Adsorption of FNIII^8-10^ on methyl surfaces in three different orientations; (**a**–**c**) “head-on”; (**d**–**f**) “beta-on”; and (**g**–**i**) “side-on”. (**a**,**d**,**g**) Snapshots at the end of simulation, (**b**,**e**,**h**) distance of the anchoring residues perpendicular to the surfaces over time, (**c**,**f**,**i**) diffusion of the anchoring residues over the surface. The colour-coding is the same as Figure 1.

When FNIII^8–10^ was inserted between two surfaces that are methyl-terminated (methyl-methyl system) we found that various initial orientations could cause different interfacial conformations after adsorption—FNd, FNa, and FNc result in “head-on”, “beta-on”, and “side-on” conformations in turn (Figure 6). The conformations “head-on”, “side-on”, and “beta-on” are respectively defined as adsorption in which the protein’s top, side, or β-sheet is in contact with the surface. However, with the initial configuration of FNb facing the hydrophobic surface, anchoring, and thus adsorption, failed repeatedly. In this configuration the cell binding site directly faces the surface. The results also suggest that when the adsorption resulted in a “head-on” conformation (Figure 6a) the cell binding and synergy sites remained exposed to the solvent, while for the other two conformations, access to the binding sites was inhibited (Figure 6d,g). Furthermore, when the final conformation was either “head-on” or “side-on” the protein fragment maintained its mobility after the anchoring (Figure 6c,i), whereas for the “beta-on” conformation, movement was highly inhibited after the anchoring (Figure 6f), suggesting stronger adsorption in this conformation.

The initial anchoring took place with the FNIII^10^ module in all cases; with Asn1457 (15.5 ns), Thr1431 (97.3 ns), and Asn1457 (43.5 ns) followed by Ser1378 (102 ns) on the other end of the protein fragment responsible for “head-on”, “beta-on”, and “side-on” adsorption processes respectively. All of the anchoring residues are polar uncharged. The residues in contact with the surface at the final stage of adsorption can be found at Table 1. Between them, twenty are polar uncharged, six are hydrophobic, two are positively charged, and one is negatively charged. Surface maps (Figure S5) reveal that a larger area of protein was in contact with the surface in the “beta-on” conformation. In fact, eleven amino acids are adsorbed onto the surface in “beta-on” conformation versus nine in “head-on” or “side-on” conformation. The maps also indicate that the area of protein in contact with the surface was mainly hydrophilic, highlighting the importance of hydrophilic residues at the early stages of adsorption.

### 2.6. Adsorption on Hydrophilic Surface

**Figure 7:**
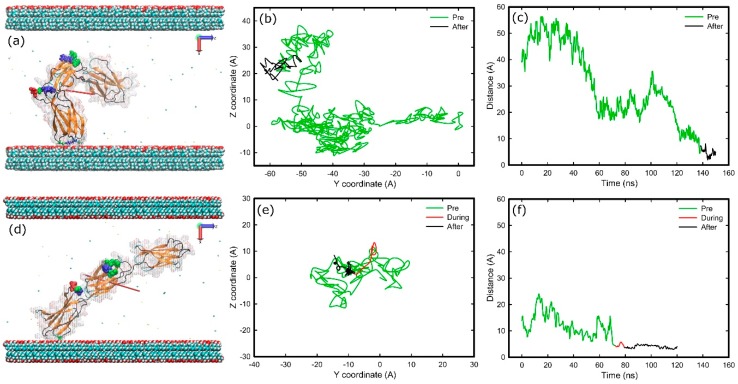
Adsorption of FNIII^8–10^ on hydroxyl surfaces on two different systems; (**a**–**c**) methyl-hydroxyl, (**d**–**f**) hydroxyl-hydroxyl. (**a**,**d**) Snapshots at the end of simulation, (**b**,**e**) diffusion of the anchoring residues over the surface, (**c**,**f**) distance of the anchoring residues perpendicular to the surfaces over time. The colour-coding is the same as Figure 1.

Final adsorption stages on methyl-hydroxyl and hydroxyl-hydroxyl surfaces are shown in Figure 7. In the methyl-hydroxyl system, only the first carbon of each SAMs molecule was fixed in space while in the other systems the first free carbons of each SAMs molecule were fixed (see Methods). The initial orientation for both systems was with side FNa (Figure 2) of the protein facing the hydroxyl surface but the initial distance between protein and surface on the methyl-hydroxyl system was double that in the hydroxyl-hydroxyl system.

In both systems, the FNIII^10^ module anchored first, with Asn1457 (138.5 ns) in the methyl-hydroxyl system, and with Thr1429 (71.7 ns) on the other. Both anchoring residues are polar uncharged. The list of the anchoring residues is shown in Table 1. Of them, four residues were polar uncharged, three were hydrophobic, and one was positively charged.

Adsorption resulted in “head-on” conformation in both cases, while the cell-binding and synergy regions remained exposed (Figure 7). In the hydroxyl-hydroxyl system, the anchoring residue has to penetrate a barrier that was not observed in the other system. Furthermore, in the methyl-hydroxyl system, the protein maintained some mobility after adsorption, as the diffusion of the protein over the surface reveals in Figure 7b, whereas protein was immobilised after adsorption in the hydroxyl-hydroxyl system (Figure 7b,e). The electrostatic maps (Figure S6) reveal that a variety of residues are in contact with the surface, while approximately eight hydrogen bonds were formed between protein and hydroxyl surface in the final stage of adsorption.

## 3. Discussion

### 3.1. Adsorption Simulations on Charged Surfaces

Simulation results confirm that electrostatics are the main driving forces for the adsorption of fibronectin fragment on the positively charged systems while in the case of negatively charged surface (carboxyl-terminated) the picture is slightly more complicated. Whilst adsorption on positively charged surfaces is fast, driven by electrostatics and results in “side-on” conformation, adsorption on the carboxyl-terminated surface is less specific and relies on the interaction of a positive side-chain from the protein surface being available to anchor the protein.

**Figure 8:**
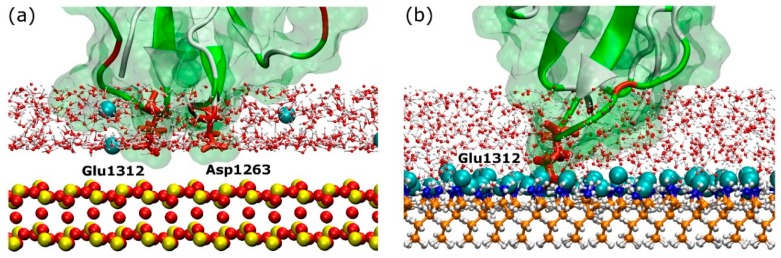
Anchoring site between FNIII^8^ module and (**a**) silica, (**b**) amine surface. Anchoring residues are shown by thick red sticks and are annotated, while the surfaces are shown by CPK representation. The colour-coding for the surface is: red–oxygen; yellow–silicon; orange–carbon; white–hydrogen; and blue–nitrogen. The teal spheres represent Cl^−^ ions.

The rapid and directed adsorption on the positively charged surfaces, unlike that on the negative carboxyl-terminated surface, is due to the negatively charged fragment being steered by the electric field. After anchoring on the model, rigid silica surface, the fragment maintains some mobility across the surface (Figure 4). In contrast, the anchoring residues were immobilised upon adsorption on amine-terminated surface. It is very likely that differences in adsorption characteristics are due to the rigidity of the substrate. Unlike the atoms that are fixed in space on the silica surface, the molecules that compose the amine surface possess a certain degree of flexibility because only the first few carbon atoms next to the underlying substrate are fixed in space. As a result, the amine surface can be considered as a “soft” surface that allows the anchoring residues to penetrate the SAMs molecules, create more bonds and, consequently, inhibit their surface movement. The explanation is supported by detailed snapshots of the negatively charged anchoring residues on the two surfaces discussed, as shown in Figure 8. The model silica surface atoms are densely packed and fixed in space, therefore, anchoring residues and ions appear at well-defined distances from the surface as determined by van der Waals radii of surface atoms. The “empty” space above silica surface in Figure 8a actually represents the van der Waals volume of surface atoms, but is not shown for clarity’s sake. In contrast, due to flexibility of the SAM molecules (and especially the terminal groups), the large free volume available at the surface allows ions, water, and anchoring residue(s) to penetrate the surface. Strong anchor–SAM surface interactions immobilise the anchor (and entire fragment). 

It is also worth noting that the anchoring residues have to overcome an energy barrier to adsorb firmly on the SAM systems. In these systems, the surface bound water is not organised in well-defined water layers as observed for the model silica, but instead forms a layer consisting of ions and water molecules strongly bound to the charged underlying substrate, which act as a barrier. To anchor successfully, the residue has to compete with the water molecules and ions for the available free volume. However, once achieved, the small distance between the anchoring residue and charged functional groups on the SAM surface results in strong adhesion that restricts the mobility of the anchoring residue (Figure 4a,c and Figure 5b).

The initial anchoring on both positively charged surfaces took place with the FNIII^8^ that was closer to the positively charged surface than the FNIII^10^ module at the initial configuration. Moreover, the overall charge of FNIII^8^ is −4 e cf zero e of the FNIII^10^ module, which could result in a stronger attractive interaction with the positively charged functional groups on the surface. Residue Thr1509 was found responsible for subsequent surface anchoring with the FNIII^10^ module. Because it is the last residue in the polypeptide chain (C-terminus), it possesses a negative charge, which causes electrostatic attraction and anchoring towards the positively charged surface. The anchoring could be further facilitated by the flexibility of the C-terminus. It is worth noting that in the case of full-length fibronectin, the Thr1509 would not be the C-terminal and possibly would not play such a significant role during anchoring. Similarly, an exposed C-terminal of a full-length protein could play a significant role during adsorption on positively charged surfaces and could be the study of another project. The adsorption results on hydrophobic, hydrophilic, and charged surfaces are summarised in Table 2.

Due to the presence of an electric field on the positively charged system, adsorption is always preceded by alignment of the protein dipole moment with the field. Given the positive patch around the cell-binding region, it remained exposed to the solvent and available for subsequent cell binding. In contrast, due to the less-specific adsorption onto the carboxyl surface, the availability of the cell-binding domain remains unclear, but the proximity of the positive patch to the cell-binding region is likely to render it unavailable.

### 3.2. Adsorption on Uncharged Surfaces

Adsorption on uncharged surfaces is mainly driven by short-range interactions. The protein fragment follows Brownian motion in the water box (free diffusion) until one of the residues comes in close proximity with the surface and anchors on it. Consequently, the protein could adopt various conformations upon the adsorption on uncharged surfaces, which is dependent on both the initial orientation of the protein against the uncharged surface and the non-specific trajectory of the protein prior to surface adsorption. 

For adsorption on a methyl-terminated surface, the initial anchoring takes place with the FNIII^10^ module, which might be because this module is more hydrophobic than module FNIII^8^, having a hydropathy index of −11 cf. −31 for FNIII^8–10^. It is worth noting that hydrophilic residues are responsible for anchoring on hydrophobic surfaces simply because they are exposed to the solute while the hydrophobic ones are usually buried. Consequently, hydrophilic residues are readily available to approach the surface and form the initial anchoring. Once the protein has adsorbed, it occupies the free volume over the surface and excludes the water molecules and ions from the protein–surface interface. As a result, the hydrophobic residues that were buried in the core of the protein tend to come closer to the methyl surface driven by the hydrophobic effect, which leads to stronger interaction between the protein and the methyl surface. However, it is not clear why polar uncharged residues, but not charged residues, are involved during the initial anchoring on methyl surfaces. It could have been a random event given that approximately a quarter of the total hydrophilic residues that compose FNIII^8–10^ are charged, which reduces the chances of anchoring with one of them. 

The adopted conformations in the final stage of adsorption onto methyl surfaces are non-specific and not affected by the initial anchoring or starting orientation. Anchoring with the residue Thr1431 results in “head-on” and “beta-on” conformations, while anchoring with the residue Asn1457 results in “head-on” and “side-on” conformations. Furthermore, the final confirmation of the fragment is not dependent on the starting orientation of the protein, because with the side FNa facing the surface, adsorption resulted in “head-on” and “beta-on” conformations. We conclude that the possibility of the cell-binding region to remain exposed after adsorption on methyl surface was irrelevant to the starting orientation or the anchoring.

In contrast to the adsorption on charged SAM surfaces, on the methyl-terminated surface FNIII^8–10^ maintained a relative mobility across the surface after adsorption, and in at least three out of four cases was not immobilised. The only exception is when the adsorbed protein adopts a “beta-on” conformation, which reduces the mobility significantly though not to the extent observed with charged surfaces. This might be because the “beta-on” conformation not only facilitates an increased number of residues adsorbed on the surface, but also excludes water molecules from the protein-surface interface, resulting in an enhanced adhesion between hydrophobic residues from the core of the protein and the methyl-terminated surface. No signs of a strongly bound layer of water molecules and ions were observed during the adsorption on methyl surfaces (unlike adsorption onto charged SAM surfaces), which was expected given that the surfaces were uncharged. This was further supported by ions being randomly dispersed in the water box and not adjacent to the surface due to the lack of electric field.

In both hydrophilic systems, the initial orientation of FNIII^8–10^ was with the side FNa facing the surface. The anchoring took place with two polar uncharged residues, with Thr1429 on the hydroxyl-hydroxyl system and Asn1457 for the methyl-hydroxyl, and both resulted in “head-on” conformations. Both the anchoring residues are present in the FNIII^10^ module of the protein, which was responsible for the anchoring onto the methyl surface as well. Therefore, it is likely that the anchoring is irrelevant to the hydrophobicity of the module and is a non-specific event that depends on the Brownian motion of the protein. Because the initial distance between FNIII^8-10^ and the hydroxylated surface of the methyl-hydroxyl system was larger than that in the other systems, the protein fragment had more space to rotate and adsorbed almost perpendicular to the surface. The anchoring was observed after 140 ns cf. 80 ns on the hydroxyl-hydroxyl surface, which further supports the role of Brownian motion.

A layer of water molecules was expected be bound onto the hydroxyl-terminated surfaces due to short-range forces (vdW), although not as firmly as on the charged surfaces (with electrostatic forces). However, the results suggest that the anchoring residue had to penetrate a barrier only during the adsorption in the hydroxyl-hydroxyl system. The only difference was the number of carbons that were kept fixed in space; on the methyl-hydroxyl system, only the first carbon atoms were fixed in space cf. the first three carbon atoms for the hydroxyl-hydroxyl system. SAMs molecules had a lower degree of freedom in the hydroxyl-hydroxyl cf. methyl-hydroxyl system. Therefore, water molecules could form a more compact layer on the methyl-hydroxyl cf. hydroxyl-hydroxyl surface, which in turn introduced a higher barrier into this system. Furthermore, it would be expected that the greater degree of flexibility in the building SAMs molecules on the methyl-hydroxyl system would have a greater impact on the mobility of the adsorbed protein, as was seen during adsorption on charged surfaces. However, the effect was opposite. It seems that reduced mobility on the hydroxyl-hydroxyl system is caused by higher number of anchors and increased interfacial tension between the hydrophobic anchoring residues and the hydroxyl surface, however a further investigation is needed [39]. Lastly, the adsorption on both hydrophilic systems resulted with the cell-binding region exposed on the solution and available for subsequent cell binding.

### 3.3. Structural Changes upon Adsorption

The RMSD values (Figure S7) show that module FNIII^9^ maintains the highest structural integrity during the simulations, possibly because it is the module in the middle and is protected by the other two modules. In contrast, module FNIII^10^ was found to have the lowest structural integrity, which could be attributed to the mobility of the loop containing the RGD site that provides flexibility to the region in order to facilitate cell binding. The modules are deformed during surface adsorption, as expected, whilst the deformation experienced on charged surfaces is nearly twice that experienced on non-charged surface. However, the RMSD values for each individual module are around two Å even after adsorption, suggesting that the modules remain intact and do not change their structure. The increased values of RMSD for the entire protein suggest that the fragment bends or twists on the loops connecting subsequent modules. The bend between subsequent modules of the protein fragment in the present MD experiments might be greater than that in the native protein due to a lack of stabilisation coming from the remaining parts of the protein. The distance between the RGD and PHSRN sites is approximately 30 Å in cases of no adsorption, but is within the range between 20 Å and 40 Å when there is surface adsorption. This could have an effect on the ability of the protein to bind with the cells and further investigation is needed. Lastly, no differences in the structural integrity of the protein fragment were observed between adsorption on hydrophilic and on hydrophobic surfaces. It is known that adsorption on hydrophobic surfaces can result in denaturation of the adsorbed proteins, however, this cannot be seen in the present study due to the time scales used in our MD simulations.

Like any other fully atomistic classical MD approach, the present work is focused on the first few tens of nanoseconds of protein adsorption on a surface, which is the first stage of protein adsorption but not the final stable state that can be different, as suggested by previous work [40]. Nevertheless, our results were found in agreement with previous experimental results, such as those presented by Michael and coworkers [35]. In their work on the adsorption of FNIII^7–10^ on SAMs surfaces a similar preference for the fragment to adsorb onto positively charged or hydrophobic surfaces cf. negatively charged or hydrophilic surfaces was found. These researchers also showed that the activity of the adsorbed fragment, which is directly related to the exposure of the cell-binding domain, was greater on positively charged or hydrophilic surfaces than on negatively charged or hydrophobic ones. The present work demonstrates that owing to the polarity of the fragment and the positive patch around the cell-binding region, the cell-binding region remains exposed upon adsorption on positively charged surfaces. At the same time, adsorption on uncharged surfaces is non-specific, whereas a stronger adsorption and denaturation on hydrophobic surface results in lower activity of the fragment, especially at low concentrations where protein–protein interactions are absent. 

## 4. Materials and Methods 

All simulations were performed with the NAMD 2.6 package, using the Charmm27 force field, while the images were analysed with the VMD software [41,42]. The fibronectin fragment FNIII^8–10^ used in the present work was extracted from the domain FNIII^7–10^ solved by Leahy et al. [43] and was downloaded from the protein database (PDB code: 1FNF). The protein fragment FNIII^8-10^ contains the residues 1236 to 1509 of the overall fibronectin sequence.

Initially, FNIII^8–10^ was placed in a rectangular box filled with water molecules (TIP3P model) that extended at least 17 Å from every protein atom, resulting in a system of approximately 100,000 atoms. To neutralise the protein fragment that has an overall charge of −5 e, NaCl ions were added to maintain an ionic strength of 0.05 M (mol/L). Subsequently, the system was set to perform a trajectory of 100 ns (the computational details are the same as for the adsorption trajectories and are given below). The structure of the fragment after 60 ns of dynamics was used as the starting structure for all of the following adsorption simulations.

During the adsorption simulations, four different arrangements were used as initial orientations—the surfaces were placed in either (x, z) or (y, z) planes, which were placed against either side of the protein. The protein-surface distance varied between seven Å and twenty Å—it was kept at approximately twenty Å against charged surfaces but below ten Å on neutral surfaces to facilitate protein adsorption. Every system had approximately 100,000 to 120,000 atoms. To carry out the protein adsorption simulation, the system was subjected to 1000 steps of water minimisation initially, followed by 100 ps of water equilibration at a temperature of 300 K. Subsequently, the system (water and protein) was minimised for 10,000 steps, before being heated up to 300 K for 45 ps, and equilibrated at the constant temperature of 300 K for 555 ps. Finally, the production of the trajectories was performed for 100 to 150 ns at the NVT ensemble. The SHAKE algorithm and periodic boundary conditions were used for the simulations. The cut off distance for the van der Waals interactions was 12 Å, while the smooth particle mesh Ewald (PME) summation was used for the electrostatic interactions [44].

### Model Surfaces

Two different types of models were used in this work; a silica surface and a surface replicating self-assembled monolayer (SAMs). The silica surface had been used in the previous work [45] from which further information can be found. In brief, the atoms of the SiO_2_ surface were fixed in space in order to represent a slab that has been cut from the bulk crystal in such a way that left siloxide (SiO^−^) groups on the top of the slab and under-coordinated silica species on the bottom. As a result, the surface has an intrinsic dipole moment across it. Because periodic conditions were used in all simulations and, owing to the overall negative charge of FNIII^8–10^, the protein fragment was adsorbed on the positively charged side of the model surface. The dimension of the surface was 70 × 145 Å.

**Figure 9:**
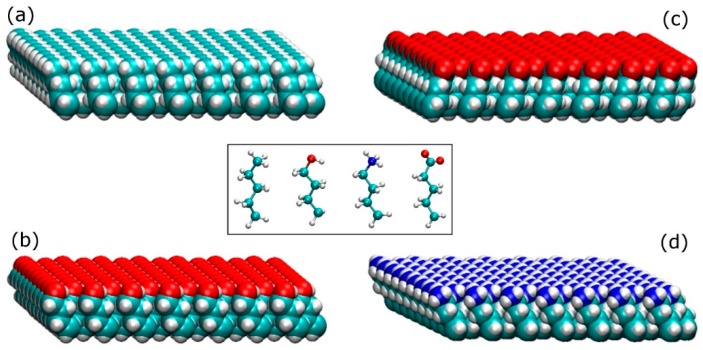
Self-assembled monolayers (SAMs)-replicating model surfaces; (**a**) methyl-terminated (−CH_3_), (**b**) hydroxyl-terminated (−OH), (**c**) carboxyl-terminated (−COO^−^), and (**d**) amine-terminated (−NH_3_^+^). On the inset are shown the building molecules for the SAM replicated model surfaces (from left to right; methyl-terminated, hydroxyl-terminated, amine-terminated, and carboxyl-terminated).

The SAMs surfaces were constructed with four different terminal functionalities; methyl (−CH_3_), hydroxyl (−OH), amine (−NH_3_^+^), and carboxyl (−COO^−^), as shown on the inset of Figure 9. These end groups represent a non-polar surface (hydrophobic), a polar uncharged surface (hydrophilic), a positively charged surface (hydrophilic), and a negatively charged surface (hydrophilic), respectively. The selected molecules were truncated from the following amino acids with the desired end group; an isoleucine (methyl group), a serine (hydroxyl group), a lysine (amine group), and an aspartic acid (carboxylic acid group). Each molecule has a backbone consisted of 4 carbon molecules in addition to the designated functional group, while the broken C–C bond was patched with hydrogen atoms to satisfy the valence requirements.

The individual molecules were placed parallel to each other with a distance of 4.97 Å between them [39] to generate the SAMs surfaces shown in Figure 9. Those basic surfaces were used in pairs (one on top of the other with the functional groups facing outside) in order to build four different systems of SAMs surfaces; (i) an amine-carboxyl, (ii) a methyl-hydroxyl, (iii) a methyl-methyl, and (iv) a hydroxyl-hydroxyl. Each of the SAMs surfaces contained 16,000 atoms and had dimensions of 140 × 75 × 14 Å. On the first system (amine-carboxyl), basic surfaces (Figure 9) containing terminal functionalities with opposite charges were placed on the two sides of the surface, resulting in a surface that had an intrinsic dipole moment perpendicular to it. Similar to the arrangement for silica surfaces described above, the 3D periodicity of the simulation box creates a force field across the water/protein medium that mimics the electric field created from charged surfaces. At pH = 7, although in a real system not all species on the amine and carboxyl terminated surfaces are charged, here we used fully charged species to accelerate the adsorption process. The first three carbon atoms (from the bottom) of every SAM molecule were fixed in space, leaving the last carbon on the backbone and the terminal functional group free to move. NaCl at a concentration of either 0.8 M or 1.0 M was introduced to the simulation box for the adsorption on the positively or the negatively charged side, respectively. In the second system (methyl–hydroxyl), basic surfaces with methyl and hydroxyl functional groups were placed on the opposing sides of a surface to build a hydrophilic-hydrophobic system. Only the first carbon of each SAM molecule was fixed in space, while the other three carbons and the end group were able to move. The final two systems have either methyl (methyl–methyl) or hydroxyl (hydroxyl–hydroxyl) on both sides of the surface whilst the first three carbons are fixed in space. Sodium chloride (0.05 M) was added in all of the uncharged systems.

## 5. Conclusions

In the present study, the effect of surface chemistry on the adsorption mechanism of the fibronectin fragment FNIII^8–10^ was investigated and the following conclusions can be drawn. When the adsorption was driven by a long-range electric field above a positive surface, as happens with the model silica and amine surfaces, the adsorption is rapid and site-specific. The dipole moment of the protein quickly aligns with the electric field and the protein is rotated, whilst the strong attraction results in a “side-on” conformation on the amine surface. Furthermore, due to a positive patch around the cell-binding site, the protein remains functional upon adsorption on amine and able for subsequent cell binding. In contrast, above a negatively charged surface with adequate ionic screening, as with our carboxyl-terminated surface, the adsorption is less specific. The anchoring residues are of opposing charge to that of the surfaces or polar uncharged, while a strongly bound layer of water molecules and ions inhibits the anchoring and adsorption of protein on charged surfaces. Furthermore, “soft” surfaces have a higher impact on the mobility of the proteins after adsorption as they highly restrict their movement.

The adsorption on uncharged surfaces is relatively slow and non-specific. The protein undertakes Brownian motion until the right residue is in the right place at the right time to facilitate the anchoring event. The anchoring residues, both for hydroxyl and methyl surfaces, are polar uncharged, highlighting their importance at the initial stages of adsorption. The conformations of the fragment upon adsorption can be “head-on”, “beta-on”, and “side-on”, and they were independent of the initial orientation of the fragment or the initial anchoring residue. The “beta-on” conformation was found to be the strongest, as it facilitates the exposure of core hydrophobic residues due to the hydrophobic effect.

## Figures and Tables

**Table 1:** Residues in contact with the surface at the final stage of adsorption.

Surface	Anchoring Residues
Silica	Ser1261, Asp1263, Thr1265, Glu1278, Asp1279, Ser1286, Ser1288, Asp1289, Tyr1311, Glu1312, Asp1377, Glu1424, Val1426, Ala1428, Thr1429, Asp1438, Pro1480, Arg1508, Thr1509
NH_3_^+^	Glu1312, Thr1509
COO^−^	Lys1469
CH_3_–OH	Pro1430, Thr1431, Ser1458, Pro1459, Lys1478, Pro1479, Gly1480
CH_3_ “head-on”	Thr1454, Gly1455, Gly1456, Asn1457, Lys1478, Pro1479, Gly1480, Val1481, Thr1509,
CH_3_ “side-on”	Lys1275, Asn1276, Thr1355, Pro1376, Ser1378, Thr1454, Gly1455, Gly1456, Asn1457
CH_3_ “beta-on”	Thr1431, Tyr1446, Asn1457, Ser1458, Pro1459, Gln1461, Phe1463, Thr1464, Pro1466, Ser1475, Gly1476
OH–OH	Thr1429, Pro1430, Thr1431, Pro1479
CH_3_–OH	Asn1457, Lys1478, Pro1479, Gly1480

**Table 2:** Number of successful adsorptions, initial anchoring residues, resulted conformation, availability for cell binding domain after adsorption, specific orientation, mobility after anchoring, and anchoring time on each type of surface.

Surface	Successful Adsorptions	Anchoring Residues	Resulted Conformation	Cell-Binding Domain	Specific Orientation	Mobile	Anchoring Time (ns)
Hydrophobic	4/14	Thr1431	Head-on	Exposed	No	Yes	35.0
		Thr1431	Beta-on	Buried	No	No	97.3
		Asn1457	Head-on	Exposed	No	Yes	15.5
		Asn1457 & Ser1378	Side-on	Buried	No	Yes	43.5
Hydrophilic	2/3	Asn1457	Head-on	Exposed	No	Yes	138.5
		Thr1429	Head-on	Exposed	No	No	71.7
+ Charged	2/2	Ser1261 & Thr1509	Side-on	Exposed	Yes	Yes	3.1
		Glu1312 & Thr1509	Side-on	Exposed	Yes	No	5.3
− Charged	1/2	Lys1469	-	Buried	No	No	53.0

## Supplementary Materials

Supplementary materials can be found at http://www.mdpi.com/1422-0067/19/11/3321/s1.
