# Supplementary material for: Adsorption of Fibronectin Fragment on Surfaces Using Fully Atomistic Molecular Dynamics Simulations
Source: Int J Mol Sci. 2018 Oct 25;19(11):3321. doi: 10.3390/ijms19113321 (PMC6275015; doi:10.3390/ijms19113321)
Supplement: Supplementary file 1 [file ijms-19-03321-s001.pdf]

Supplementary Materials:

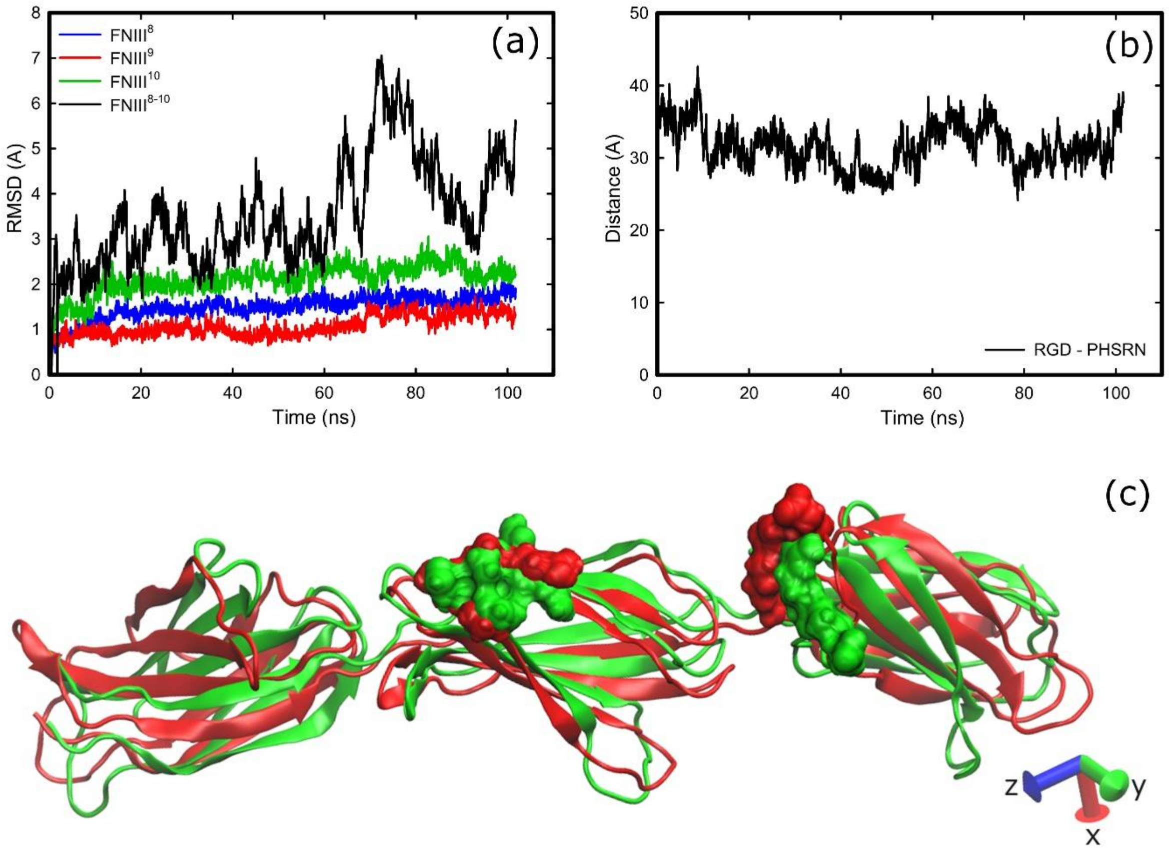

Figure S1. (a) RMSD calculated for the trajectory of FNIII$^{8-10}$, (b) evolution of distance between RGD and PHSRN sites during the trajectory of FNIII$^{8-10}$, (c) structural difference of FNIII$^{8-10}$ at t=0 ns (green) and t=60 ns (red).

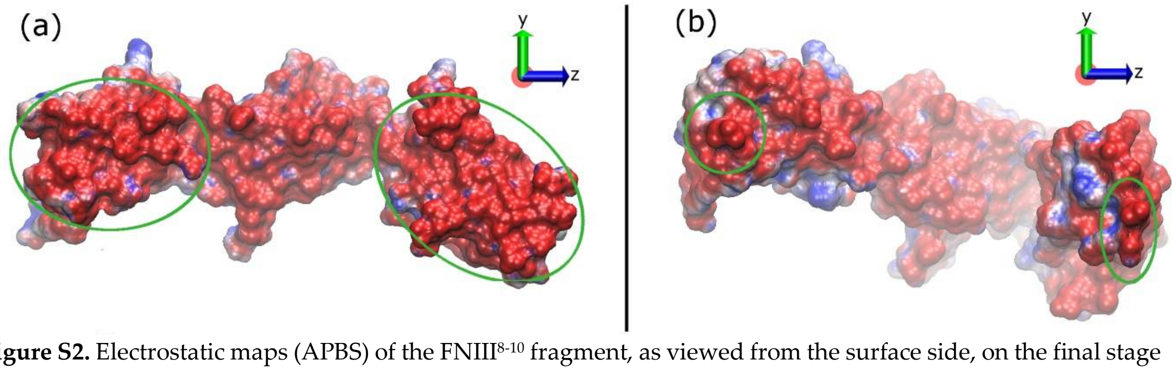

Figure S2. Electrostatic maps (APBS) of the FNIII$^{8-10}$ fragment, as viewed from the surface side, on the final stage of adsorption onto silica (a) and amine (b) surfaces. The surface atoms are not shown for clarity. The deep cueing option of VMD is being used to indicate the distance of each atom from the surface. The areas inside the green circles indicate the areas that are in contact with the surface. The colour scheme is the same as in Figure 2.

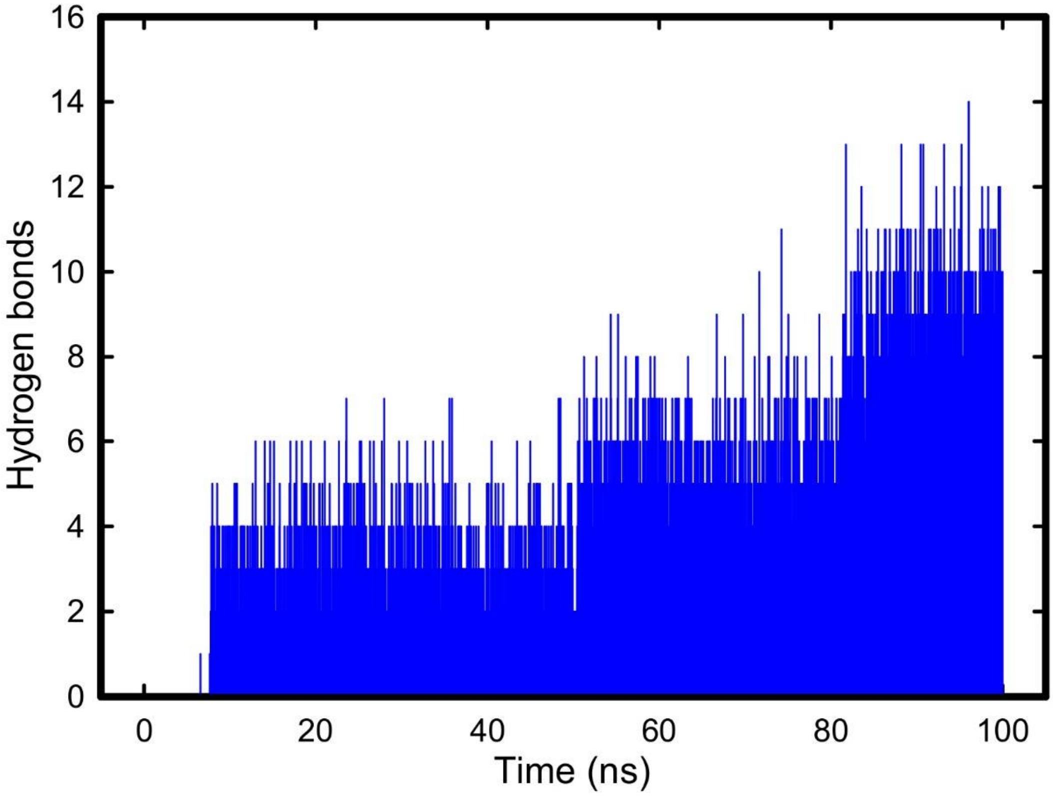

Figure S3. Evolution of the number of H-bonds between FNIII$^{8-10}$ and the amine surface over time.

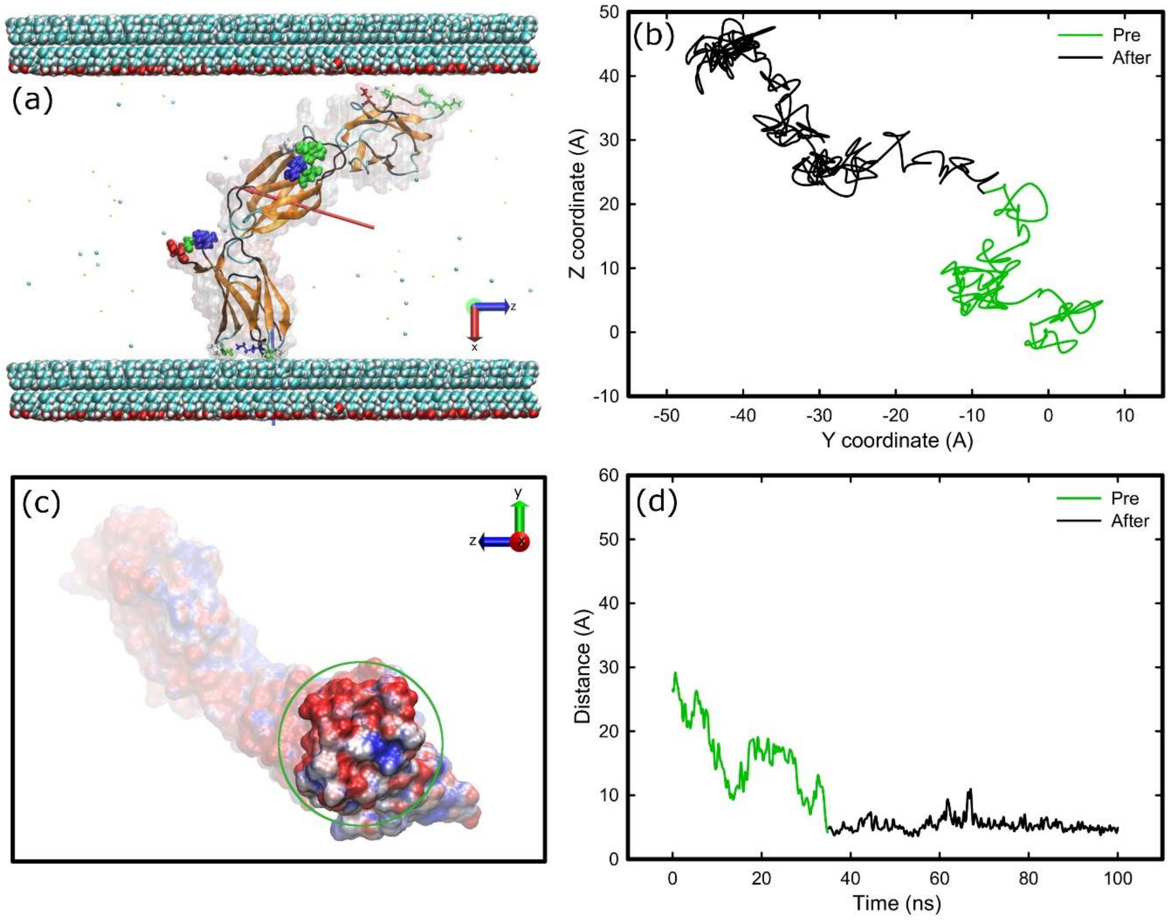

Figure S4. Adsorption of FNIII$^{8-10}$ on a methyl surface (methyl-hydroxyl system); (a) snapshot at the end of simulation, (b) diffusion of the anchoring residue over the surface, (c) electrostatic representations (APBS) of the FNIII$^{8-10}$ fragment as viewed from the methyl surface on the final stage of adsorption on the methyl-hydroxyl

system, and (d) distance of the anchoring residue perpendicular to the surface over time. The areas inside the green circles indicate the areas that are in contact with the surface. The deep cueing option of VMD is being used to indicate the distance of each atom from the surface. The colour-scheme is the same as previously, while the surface atoms and water molecules are not shown for clarity.

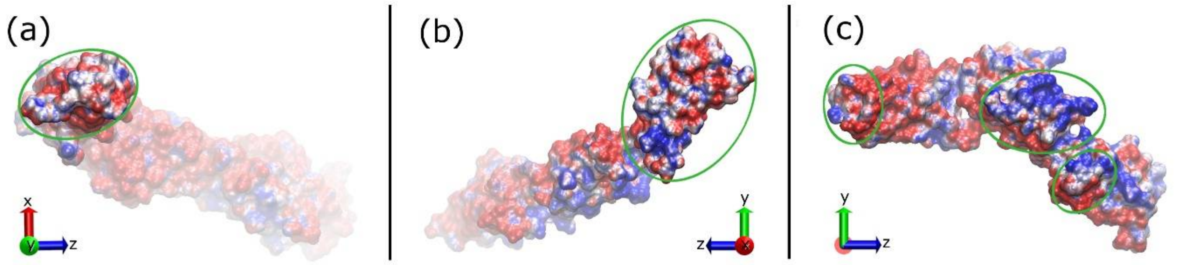

Figure S5. Electrostatic (APBS) maps of the FNIII$^{8-10}$ fragment, as viewed from the surface side, on the final stage of adsorption on methyl surfaces on three different orientations; (a) “head-on”, (b) “beta-on”, (c) “side-on”. The areas inside the green circles indicate the areas that are in contact with the surface. The deep cueing option of VMD is being used to indicate the distance of each atom from the surface. The colour-scheme is the same as previously, while the surface atoms and water molecules are not shown for clarity.

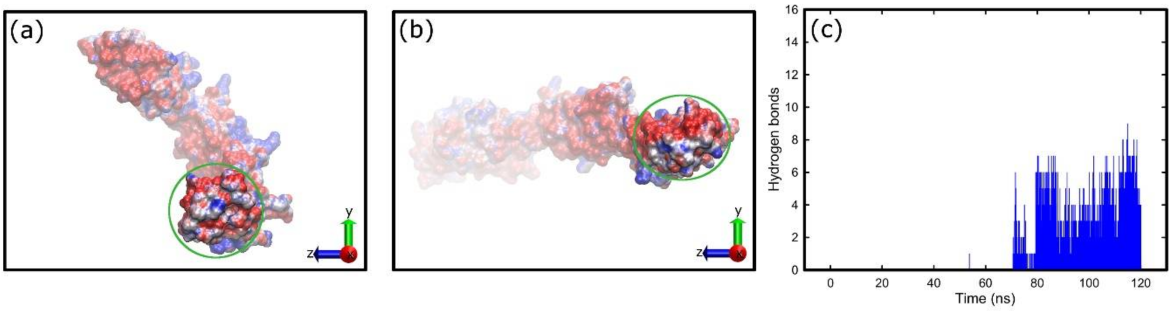

Figure S6. Electrostatic (APBS) maps of the FNIII$^{8-10}$ fragment, as viewed from the surface side, on the final stage of adsorption onto hydroxyl surfaces on two different systems; (a) methyl-hydroxyl, (c) hydroxyl-hydroxyl, and (c) evolution of the number of hydrogen bonds between FNIII$^{8-10}$ and the hydroxyl surface over time. The areas inside the green circles indicate the areas that are in contact with the surface. The deep cueing option of VMD is being used to indicate the distance of each atom from the surface. The colour-scheme is the same as previously, while the surface atoms and water molecules are not shown for clarity.

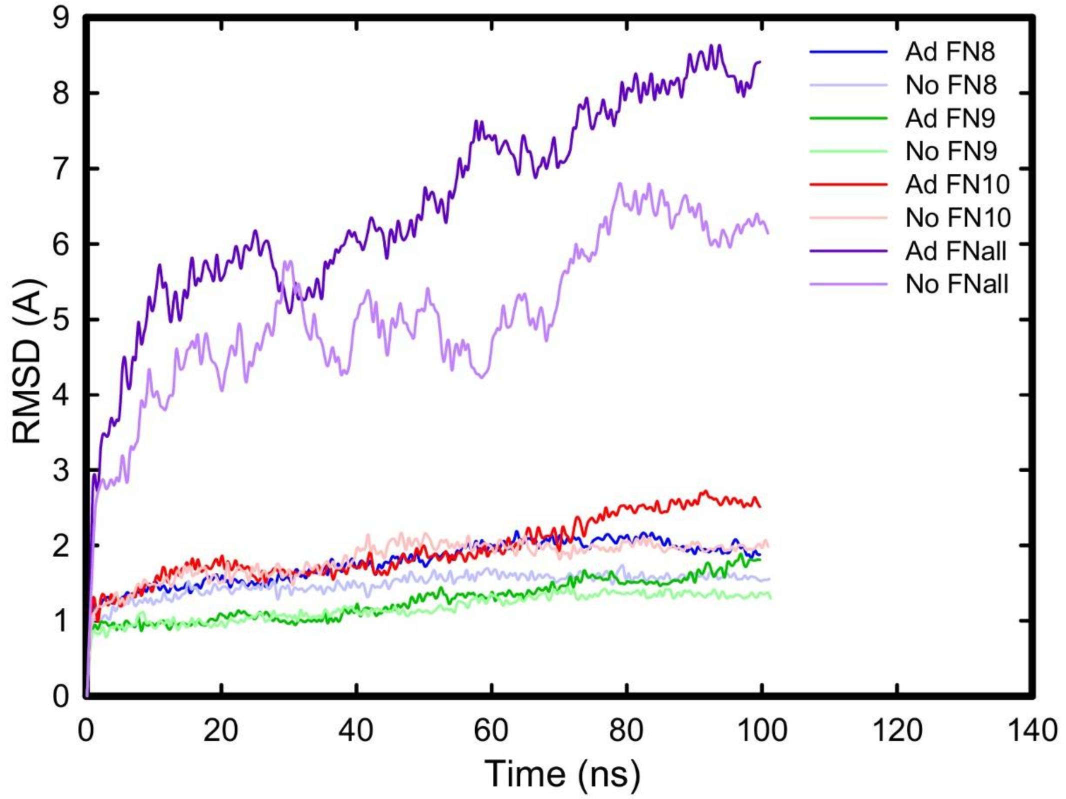

Figure S7. Average RMSD values for the individual modules (FNIII^{8}-FNIII^{10}) and for the whole fragment (FNIII$^{8-10}$), in cases of adsorption (Ad) and no adsorption (No) onto surfaces.

Table S1. Charged and hydrophobic residues of individual domains and full-length FNIII$^{8-10}$ fragment. The last two columns show the total charge and hydropathy index of the domains, while the rest of the columns show the number of residues per labelled characteristic [47].

domain	residues	hydrophobic	hydrophilic	charged	positive	negative	Total charge	hydropathy
FNIII^{8}	91	28	63	18	11	7	-4	-31
FNIII^{9}	89	26	63	17	9	8	-1	-40.4
FNIII^{10}	94	29	65	16	8	8	0	-10.8
FNIII$^{8-10}$	274	83	191	51	28	23	-5	-82.2
RGD	3	0	3	2	1	1	0	-8.4
PHSRN	5	0	5	1	1	0	1	-13.6
